# Primary Localized Amyloidosis of the Urinary Bladder Mimicking Urothelial Carcinoma: A Case Report

**DOI:** 10.7759/cureus.110914

**Published:** 2026-06-15

**Authors:** Gassan M Salih, Saad Masood, Filip Kondylis

**Affiliations:** 1 Urology, York and Scarborough Teaching Hospitals NHS Foundation Trust, York, GBR; 2 General Surgery, Mayo Hospital, Lahore, PAK

**Keywords:** bladder amyloidosis, localized amyloidosis, transurethral resection, urinary bladder lesion, visible haematuria

## Abstract

Primary localized amyloidosis of the urinary bladder is a rare benign condition that can closely mimic urothelial carcinoma clinically and cystoscopically, particularly in patients presenting with visible hematuria. We report the case of a 79-year-old gentleman with persistent visible hematuria who underwent flexible cystoscopy, computed tomography urography, transurethral resection of bladder tumor, histopathological assessment, thoracic imaging, and hematological evaluation, including serum free light chain analysis. Cystoscopy revealed a suspicious bladder lesion concerning for malignancy, but histopathology demonstrated localized amyloid deposition within the bladder wall without evidence of urothelial carcinoma. Subsequent imaging and hematological work-up showed no evidence of systemic amyloidosis, and multidisciplinary review supported a diagnosis of primary localized bladder amyloidosis. The patient recovered without perioperative complications and was advised long-term cystoscopic surveillance because of the risk of recurrence. This case highlights localized bladder amyloidosis as an important differential diagnosis for persistent visible hematuria and suspicious bladder lesions, emphasizing the need for histopathological confirmation and exclusion of systemic disease to avoid unnecessary radical intervention while ensuring appropriate follow-up.

## Introduction

Amyloidosis is a disorder characterized by extracellular deposition of insoluble fibrillar proteins within tissues and organs, resulting in variable clinical manifestations depending on the site and extent of involvement [1]. Although systemic amyloidosis commonly affects organs such as the kidneys, heart, and liver, localized amyloidosis of the urinary bladder is rare and represents an uncommon benign entity in urological practice [2]. Patients typically present with visible hematuria, irritative lower urinary tract symptoms, or both. However, its clinical and cystoscopic appearance may closely resemble urothelial carcinoma, making accurate diagnosis difficult before histopathological evaluation [3].

Given its rarity and close resemblance to bladder malignancy, localized bladder amyloidosis remains an important differential diagnosis in patients presenting with persistent hematuria and suspicious bladder lesions. Histopathological confirmation is essential to establish the diagnosis, exclude malignant pathology, and guide appropriate management. Evaluation for systemic amyloidosis is also necessary because localized and systemic forms differ substantially in prognosis, treatment, and follow-up requirements. We report this case to highlight the diagnostic challenge of primary localized bladder amyloidosis, a rare benign mimic of urothelial carcinoma, and to emphasize the importance of histopathological confirmation and exclusion of systemic amyloidosis in patients presenting with persistent visible hematuria and suspicious bladder lesions. This case report has been prepared in accordance with the Surgical CAse REport (SCARE) guidelines [4].

## Case presentation

A 79-year-old gentleman was referred to the urology service with persistent visible hematuria. He denied significant dysuria, flank pain, constitutional symptoms, or recent urinary tract infection. His medical history was notable for atrial fibrillation, managed with edoxaban, and hypertension. He was functionally independent, with a performance status of 0 and an American Society of Anesthesiologists (ASA) grade of 2. Initial clinical assessment considered both anticoagulation-related hematuria and underlying urological pathology as potential causes, particularly given the persistent nature of his symptoms.

Flexible cystoscopy demonstrated an irregular, non-papillary, suspicious lesion involving the bladder wall, with a broad-based appearance rather than a discrete papillary tumor on a narrow stalk. The lesion raised concern for urothelial malignancy, including a flat or sessile neoplastic process. Subsequent computed tomography (CT) urography showed no upper urinary tract obstruction, hydronephrosis, or radiological evidence of metastatic disease. In view of the cystoscopic findings, the patient underwent transurethral resection of bladder tumor (TURBT) in April 2025 for definitive diagnosis and management.

**Figure 1:**
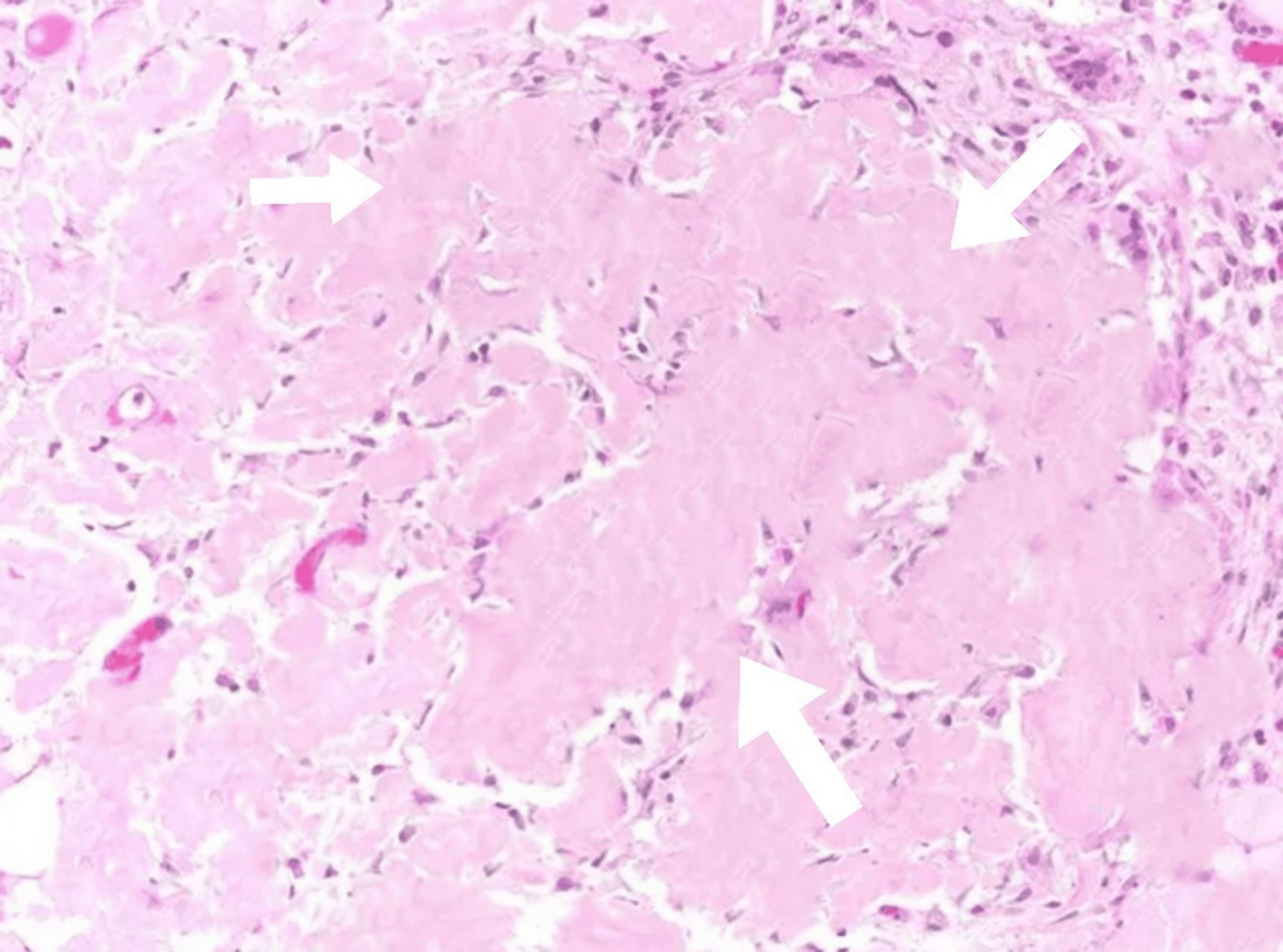
Histopathological section of the bladder wall showing amorphous eosinophilic extracellular deposits within the lamina propria, consistent with amyloid deposition (arrows). Original magnification ×200.

Histopathological examination of the resected specimen demonstrated amorphous eosinophilic extracellular deposits within the bladder wall. Congo red staining confirmed amyloid deposition, demonstrating congophilic deposits with characteristic birefringence under polarized light. There was no evidence of urothelial carcinoma or other malignant pathology in the examined tissue (Figure 1). Amyloid subtyping was not performed on the available tissue. Following confirmation of amyloid deposition, further laboratory and radiological investigations were performed to exclude infection, renal involvement, plasma cell dyscrasia, and systemic amyloidosis. CT thoracic imaging showed no features suggestive of systemic amyloid involvement or associated malignancy, while laboratory evaluation demonstrated preserved renal function, absence of proteinuria, no significant inflammatory response, negative urine culture, no monoclonal band on serum protein electrophoresis, and a normal serum free light chain ratio. Cardiac echocardiography, abdominal fat pad biopsy, and bone marrow biopsy were not performed because there were no clinical features of cardiac involvement, no renal impairment or proteinuria, no monoclonal protein, and no abnormal serum free light chain ratio to suggest systemic amyloid light-chain (AL) amyloidosis or an underlying plasma cell dyscrasia. Following review at the hematology multidisciplinary team meeting, the overall clinicopathological and investigative findings were considered most consistent with primary localized amyloidosis of the urinary bladder. The patient’s relevant laboratory investigations and systemic amyloidosis work-up are summarized in Table 1.

**Table 1:** Relevant laboratory investigations and systemic amyloidosis work-up. eGFR: estimated glomerular filtration rate; CRP: C-reactive protein; AL: amyloid light-chain.

Investigation	Patient value	Reference range	Clinical interpretation
Hemoglobin	12.8 g/dL	13.0-17.0 g/dL	Mild reduction, compatible with persistent visible hematuria but without severe anemia
White blood cell count	7.4 × 10⁹/L	4.0-11.0 × 10⁹/L	No leukocytosis to suggest active infection or systemic inflammatory process
Platelet count	238 × 10⁹/L	150-400 × 10⁹/L	Normal platelet count, with no thrombocytopenia contributing to bleeding
Serum creatinine	88 µmol/L	60-110 µmol/L	Preserved renal function
Estimated glomerular filtration rate	76 mL/min/1.73 m²	>60 mL/min/1.73 m²	No significant renal impairment or evidence of renal amyloid involvement
C-reactive protein	4 mg/L	<5 mg/L	No significant systemic inflammatory response
Urine dipstick	Blood 3+, protein negative, nitrite negative, leukocyte esterase negative	Negative	Confirms hematuria without supportive evidence of urinary tract infection
Urine culture	No significant bacterial growth	No growth	No evidence of active urinary tract infection
Serum albumin	39 g/L	35-50 g/L	Normal albumin, with no supportive evidence of nephrotic-range systemic involvement
Serum calcium	2.31 mmol/L	2.15-2.60 mmol/L	Normal calcium, with no supportive evidence of plasma cell dyscrasia
Serum protein electrophoresis	No monoclonal band detected	No monoclonal band	No biochemical evidence of monoclonal gammopathy
Serum free kappa light chains	17.8 mg/L	3.3-19.4 mg/L	Within normal range
Serum free lambda light chains	14.6 mg/L	5.7-26.3 mg/L	Within normal range
Kappa/lambda free light chain ratio	1.22	0.26-1.65	Normal ratio, arguing against AL amyloidosis or plasma cell dyscrasia

The patient recovered without perioperative complications. Given the recognized risk of local recurrence reported in the literature, ongoing cystoscopic surveillance was recommended following multidisciplinary evaluation.

## Discussion

Primary localized amyloidosis of the urinary bladder is an uncommon benign condition characterized by extracellular deposition of amyloid protein within the bladder wall in the absence of systemic disease involvement. Although amyloidosis may occur as part of systemic disease involving organs such as the kidneys, heart, liver, nerves, or haematological system, localized bladder involvement represents a distinct clinicopathological entity with different diagnostic and prognostic implications [1,2]. Its clinical importance lies in its ability to mimic urothelial carcinoma closely. Similar to previously reported cases, the present patient developed visible hematuria and had a suspicious cystoscopic bladder lesion, a pattern commonly seen in localized bladder amyloidosis, in which erythematous, nodular, or tumor-like lesions may be difficult to distinguish from malignancy on visual inspection alone [5-7].

Comparison with previously published cases highlights the typical features and diagnostic challenge of this presentation. Varghese et al. described localized primary bladder amyloidosis as a rare urological mimic of malignancy, emphasizing that suspicious cystoscopic findings may lead to initial concern for bladder cancer despite ultimately benign histopathology [5]. Other published cases have similarly shown that localized bladder amyloidosis may present as a suspicious bladder lesion requiring tissue diagnosis to distinguish it from urothelial carcinoma and other more common urological conditions [6,7]. Lee et al. further showed that urinary tract amyloidosis may resemble inflammatory conditions such as chronic cystitis, illustrating the broad differential diagnosis and nonspecific clinical appearance of this entity [3]. The present case is consistent with these reports because cystoscopy raised concern for urothelial carcinoma, but transurethral resection of bladder tumor (TURBT) histopathology demonstrated amyloid deposition without malignant pathology.

This case also adds an important practical lesson regarding hematuria assessment in older patients receiving anticoagulation. The patient was taking edoxaban for atrial fibrillation, which could have encouraged premature attribution of visible hematuria to medication-related bleeding. However, persistent visible hematuria should not be assumed to be anticoagulation-related without appropriate urological evaluation, particularly in elderly patients in whom structural pathology and urothelial malignancy remain important considerations [6]. In this context, anticoagulation should be viewed as a potential confounder rather than a sufficient explanation. The case therefore reinforces the need to avoid anchoring bias and to pursue cystoscopic and radiological assessment when hematuria is persistent, recurrent, or otherwise clinically unexplained.

Histopathology remains the decisive step in differentiating localized bladder amyloidosis from urothelial carcinoma. Radiological imaging and cystoscopic appearance can define lesion extent and assess the upper urinary tract, but they cannot reliably establish the diagnosis of amyloid deposition [8,9]. In reported cases, diagnosis has similarly depended on biopsy or resection specimens showing amorphous eosinophilic extracellular material within the bladder wall, with Congo red staining demonstrating characteristic birefringence where performed [7,9]. Qiu et al. also highlighted that pathological interpretation may occasionally be challenging, including cases with negative postoperative Congo red staining, which underscores the importance of careful clinicopathological correlation rather than reliance on a single diagnostic feature alone [7]. In the present case, TURBT provided both diagnostic tissue and local treatment, while the absence of urothelial carcinoma helped prevent unnecessary radical intervention.

Following identification of amyloid deposition, exclusion of systemic amyloidosis is essential because localized and systemic forms differ substantially in prognosis, management, and follow-up requirements [1,2]. Locke and Nieto emphasized that AL amyloidosis is a systemic plasma cell-related disorder requiring haematological evaluation and disease-specific treatment, whereas localized bladder amyloidosis is generally managed with local control and surveillance [2]. In this patient, preserved renal function, absence of proteinuria, normal serum free light chain ratio, absence of a monoclonal band, and unremarkable thoracic imaging supported localized rather than systemic disease. Although localized bladder amyloidosis is considered benign, recurrence has been described, including recurrent localized disease reported by Kelsey et al., supporting the need for long-term cystoscopic surveillance [8]. Because standardized surveillance intervals remain uncertain due to limited prospective data, follow-up should be individualized according to symptoms, cystoscopic findings, recurrence risk, and multidisciplinary judgment [8,9]. Overall, this case aligns with the existing literature while emphasizing a practical diagnostic sequence: persistent visible hematuria should prompt urological evaluation, suspicious bladder lesions require histopathological confirmation, and amyloid deposition should be followed by systematic exclusion of systemic disease before diagnosing primary localized bladder amyloidosis.

## Conclusions

Localized amyloidosis of the urinary bladder is a rare but important differential diagnosis in patients presenting with persistent visible hematuria and suspicious bladder lesions. This case highlights the diagnostic challenge created by its close clinical and cystoscopic resemblance to urothelial carcinoma, particularly in elderly patients with potential confounding factors such as anticoagulation therapy. Definitive diagnosis requires histopathological confirmation, followed by appropriate evaluation to exclude systemic amyloidosis. Recognition of this entity is clinically important because it may prevent unnecessary radical intervention while supporting appropriate long-term cystoscopic surveillance due to the potential for recurrence.
